# [4-(Methoxy­carbon­yl)benz­yl]triphenyl­phospho­nium bromide hemihydrate

**DOI:** 10.1107/S1600536808000184

**Published:** 2008-01-11

**Authors:** Saba Nazir, M. Khawar Rauf, Masahiro Ebihara, Shahid Hameed

**Affiliations:** a Department of Chemistry, Quaid-i-Azam University, Islamabad 45320, Pakistan; b Department of Chemistry, Faculty of Engineering, Gifu University, Yanagido, Gifu 501-1193, Japan

## Abstract

In the crystal structure of the title compound, C_27_H_24_O_2_P^+^·Br^−^·0.5H_2_O, there are inter­molecular O—H⋯Br hydrogen bonds between the H atoms of the water of crystallization and the bromide anions. The three phenyl rings of the triphenyl­phosphonium moiety are at angles of 59.73 (15), 79.15 (14) and 82.81 (17)° with the C/P/C planes.

## Related literature

For related literature, see: Ahmed *et al.* (1996 ▶); Harcken & Martin (2001 ▶); Kojima *et al.* (2002 ▶); McDonald & Campbell (1959 ▶); Nassar *et al.* (2004 ▶); Phillips *et al.* (2002 ▶); Tanaka *et al.* (2003 ▶); Wittig & Schöllkopf (1954 ▶).
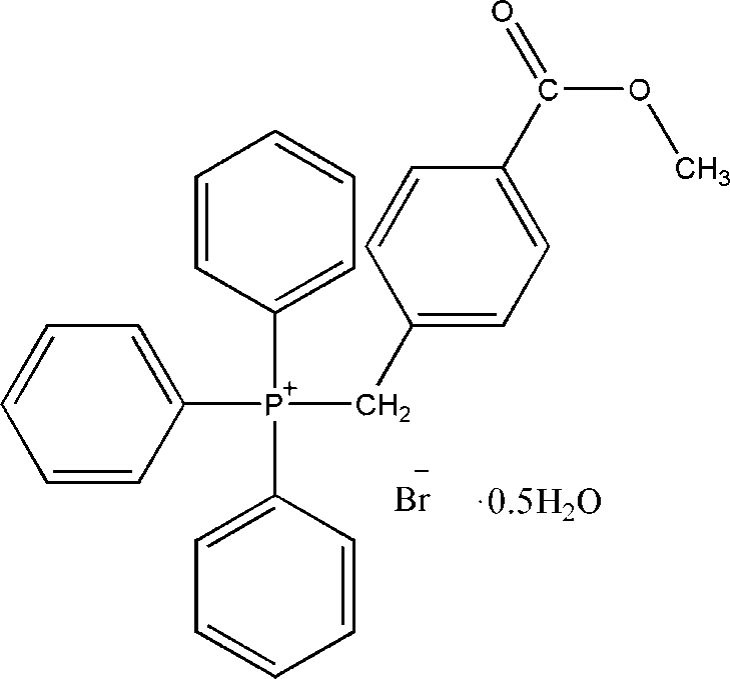


## Experimental

### 

#### Crystal data

C_27_H_24_O_2_P^+^·Br^−^·0.5H_2_O
*M* *_r_* = 500.35
Monoclinic, 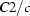

*a* = 21.017 (8) Å
*b* = 14.045 (5) Å
*c* = 19.868 (7) Å
β = 126.107 (4)°
*V* = 4738 (3) Å^3^
*Z* = 8
Mo *K*α radiation
μ = 1.83 mm^−1^
*T* = 123 (2) K
0.40 × 0.31 × 0.22 mm

#### Data collection

Rigaku/MSC Mercury CCD diffractometer
Absorption correction: integration (*ABSCOR*; Higashi, 1999 ▶) *T*_min_ = 0.404, *T*_max_ = 0.520
18847 measured reflections
5425 independent reflections
5022 reflections with *I* > 2σ(*I*)
*R*_int_ = 0.038

#### Refinement

*R*[*F* ^2^ > 2σ(*F* ^2^)] = 0.043
*wR*(*F* ^2^) = 0.079
*S* = 1.15
5425 reflections
290 parameters
H atoms treated by a mixture of independent and constrained refinement
Δρ_max_ = 0.51 e Å^−3^
Δρ_min_ = −0.53 e Å^−3^

### 

Data collection: *CrystalClear* (Molecular Structure Corporation & Rigaku, 2001 ▶); cell refinement: *CrystalClear*; data reduction: *TEXSAN* (Molecular Structure Corporation & Rigaku, 2004 ▶); program(s) used to solve structure: *SIR97* (Altomare *et al.*, 1999 ▶); program(s) used to refine structure: *SHELXL97* (Sheldrick, 2008 ▶); molecular graphics: *ORTEPII* (Johnson, 1976 ▶); software used to prepare material for publication: *SHELXL97* and *TEXSAN*.

## Supplementary Material

Crystal structure: contains datablocks I, global. DOI: 10.1107/S1600536808000184/hg2361sup1.cif

Structure factors: contains datablocks I. DOI: 10.1107/S1600536808000184/hg2361Isup2.hkl

Additional supplementary materials:  crystallographic information; 3D view; checkCIF report

## Figures and Tables

**Table 1:** Hydrogen-bond geometry (Å, °)

*D*—H⋯*A*	*D*—H	H⋯*A*	*D*⋯*A*	*D*—H⋯*A*
O3—H3*O*⋯Br1	1.03 (4)	2.22 (4)	3.2308 (17)	169 (3)

**Fig. 1.**
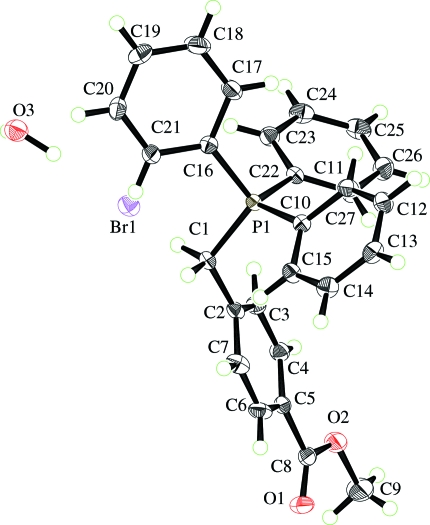
Molecular structure of (I) showing atom-labelling scheme and displacement ellipsoids at the 30% probability level.

## supplementary crystallographic information

### Comment

One of the most useful methods for the synthesis of alkenes with control over the stereoselectivity is the well known Wittig reaction (Wittig & Schöllkopf, 1954). It is mainly due to the stereoselectivity of the Wittig reagent that not only its use in the synthesis (Kojima *et al.*, 2002; Phillips *et al.*, 2002; Harcken & Martin, 2001) has not seen a decline but new and improved methods for its synthesis are being constantly developed (Nassar *et al.*, 2004; Tanaka *et al.*, 2003; Kojima *et al.*, 2002). The title compound, (I), is an intermediate in the synthesis of (*E*)-hydroxyalkyl 4-(4-substituted styryl)benzoate as a part of the project to synthesize ligands for polymeric liquid crystals. Here, we are going to present the crystal structure of the title compound (I). All the geometric parameters are in agreement with the the similar type of studies made by (Ahmed *et al.*, 1996) There are standard electrostatic interactions between the triphenyl-(4-methylcarboxy)benzylphosphonium cations and the bromide anions. It is confirmed that the compound (I) is an ion pair (Fig. 1), with a distance of 4.382 (2)Å between the P^+^ and Br^-^ centres.

### Experimental

The triphenyl-(4-methylcarboxy)benzylphosphonium bromide (I) was synthesized following a method reported in the literature (Mcdonald & Campbell, 1959).A mixture of methyl 4-(bromomethyl)benzoate 2.5 g, 0.01 mol) and triphenylphosphine (2.43 g, 0.01 mol) in 40 ml of toluene was heated under reflux for 3 hr. After cooling to room temperature the salt was filtered, washed with ether and dried under reduced pressure.Yield: 81%, m.p: 245–248°C, *R*_f_ = 0.11 (n-Hexane: ethylacetate 7:3). IR (KBr, ν_max_, cm^-1^): 3010, 2926, 2830, 1719, 1605, 786. ^1^H-NMR (CDCl_3_): δ 3.87 (3*H*, s), 5.65 (2*H*, d, *J* = 15 Hz), 7.70 (17*H*, m), 8.04 (2*H*, d, *J* = 8.1 Hz). ^13^C-NMR (75 MHz, CDCl_3_):δ 52.23, 30.58 (d, *J* = 186 Hz), 117.56 (d, *J* = 342 Hz),129.70 (d, *J* = 12 Hz), 129.87 (d, *J* = 18 Hz), 130.165 (d, *J* = 51 Hz), 131.73 (d, *J* = 21 Hz), 132.80 (d, *J* = 33 Hz), 134.45 (d, *J* = 36 Hz), 166.54.

### Refinement

H atom on the N atom was refined isotropically. Other H atoms were placed in idealized positions and treated as riding atoms with C—H distance in the range 0.95–0.99 Å and *U*_iso_(H) = 1.2*U*_eq_(C) or 1.5*U*_eq_(C).

### Crystal data

**Table:**

C_27_H_24_O_2_P^+^·Br^–^·0.5H_2_O	*F*_000_ = 2056
*M**_r_* = 500.35	*D*_x_ = 1.403 Mg m^−^^3^
Monoclinic, *C*2/*c*	Mo *K*α radiation λ = 0.71070 Å
Hall symbol: -C 2yc	Cell parameters from 7012 reflections
*a* = 21.017 (8) Å	θ = 3.1–27.5º
*b* = 14.045 (5) Å	µ = 1.83 mm^−^^1^
*c* = 19.868 (7) Å	*T* = 123 (2) K
β = 126.107 (4)º	Block, colorless
*V* = 4738 (3) Å^3^	0.40 × 0.31 × 0.22 mm
*Z* = 8	

### Data collection

**Table:**

Rigaku/MSC Mercury CCD diffractometer	5022 reflections with *I* > 2σ(*I*)
Monochromator: graphite	*R*_int_ = 0.038
*T* = 123(2) K	θ_max_ = 27.5º
ω scans	θ_min_ = 3.2º
Absorption correction: integration(ABSCOR; Higashi, 1999)	*h* = −27→25
*T*_min_ = 0.404, *T*_max_ = 0.520	*k* = −18→17
18847 measured reflections	*l* = −18→25
5425 independent reflections	

### Refinement

**Table:**

Refinement on *F*^2^	Secondary atom site location: difference Fourier map
Least-squares matrix: full	Hydrogen site location: inferred from neighbouring sites
*R*[*F*^2^ > 2σ(*F*^2^)] = 0.043	H atoms treated by a mixture of independent and constrained refinement
*wR*(*F*^2^) = 0.079	*w* = 1/[σ^2^(*F*_o_^2^) + (0.0133*P*)^2^ + 10.1118*P*] where *P* = (*F*_o_^2^ + 2*F*_c_^2^)/3
*S* = 1.15	(Δ/σ)_max_ = 0.001
5425 reflections	Δρ_max_ = 0.51 e Å^−^^3^
290 parameters	Δρ_min_ = −0.53 e Å^−^^3^
Primary atom site location: structure-invariant direct methods	Extinction correction: none

### Special details

**Table:**

Geometry. All e.s.d.'s (except the e.s.d. in the dihedral angle between two l.s. planes) are estimated using the full covariance matrix. The cell e.s.d.'s are taken into account individually in the estimation of e.s.d.'s in distances, angles and torsion angles; correlations between e.s.d.'s in cell parameters are only used when they are defined by crystal symmetry. An approximate (isotropic) treatment of cell e.s.d.'s is used for estimating e.s.d.'s involving l.s. planes.
Refinement. Refinement of *F*^2^ against ALL reflections. The weighted *R*-factor *wR* and goodness of fit *S* are based on *F*^2^, conventional *R*-factors *R* are based on *F*, with *F* set to zero for negative *F*^2^. The threshold expression of *F*^2^ > σ(*F*^2^) is used only for calculating *R*-factors(gt) *etc*. and is not relevant to the choice of reflections for refinement. *R*-factors based on *F*^2^ are statistically about twice as large as those based on *F*, and *R*- factors based on ALL data will be even larger.

### Fractional atomic coordinates and isotropic or equivalent isotropic displacement parameters (Å^2^)

**Table:**

	*x*	*y*	*z*	*U*_iso_*/*U*_eq_	
P1	0.31029 (3)	0.44471 (4)	0.32117 (3)	0.01545 (12)	
C1	0.41411 (12)	0.41713 (15)	0.38823 (13)	0.0174 (4)	
H1A	0.4424	0.4655	0.3789	0.021*	
H1B	0.4324	0.4234	0.4468	0.021*	
C2	0.43759 (12)	0.32001 (16)	0.37752 (12)	0.0181 (4)	
C3	0.42000 (13)	0.23839 (16)	0.40364 (13)	0.0206 (5)	
H3	0.3909	0.2438	0.4261	0.025*	
C4	0.44456 (13)	0.14960 (17)	0.39714 (14)	0.0222 (5)	
H4	0.4314	0.0945	0.4143	0.027*	
C5	0.48845 (12)	0.14061 (16)	0.36557 (13)	0.0205 (5)	
C6	0.50870 (13)	0.22217 (18)	0.34252 (14)	0.0249 (5)	
H6	0.5408	0.2170	0.3235	0.030*	
C7	0.48255 (13)	0.31103 (18)	0.34700 (14)	0.0241 (5)	
H7	0.4953	0.3660	0.3292	0.029*	
C8	0.51658 (13)	0.04628 (18)	0.35787 (14)	0.0260 (5)	
O1	0.56546 (10)	0.03619 (14)	0.34485 (11)	0.0341 (4)	
O2	0.48183 (10)	−0.02626 (12)	0.36867 (12)	0.0343 (4)	
C9	0.50683 (18)	−0.1213 (2)	0.3647 (2)	0.0495 (8)	
H9A	0.4922	−0.1329	0.3085	0.074*	
H9B	0.4811	−0.1685	0.3776	0.074*	
H9C	0.5641	−0.1266	0.4052	0.074*	
C10	0.27533 (12)	0.45605 (15)	0.21423 (13)	0.0167 (4)	
C11	0.19410 (13)	0.45949 (17)	0.15136 (13)	0.0217 (5)	
H11	0.1578	0.4523	0.1648	0.026*	
C12	0.16704 (14)	0.47353 (17)	0.06923 (14)	0.0252 (5)	
H12	0.1120	0.4762	0.0265	0.030*	
C13	0.21980 (14)	0.48367 (16)	0.04915 (14)	0.0240 (5)	
H13	0.2008	0.4929	−0.0072	0.029*	
C14	0.30000 (14)	0.48039 (17)	0.11111 (14)	0.0243 (5)	
H14	0.3361	0.4875	0.0974	0.029*	
C15	0.32787 (13)	0.46655 (16)	0.19402 (13)	0.0212 (5)	
H15	0.3830	0.4643	0.2366	0.025*	
C16	0.29724 (13)	0.55555 (15)	0.35705 (13)	0.0182 (4)	
C17	0.22172 (14)	0.58176 (18)	0.33081 (17)	0.0311 (6)	
H17	0.1780	0.5419	0.2939	0.037*	
C18	0.21096 (16)	0.66619 (19)	0.35887 (18)	0.0363 (6)	
H18	0.1596	0.6842	0.3409	0.044*	
C19	0.27431 (15)	0.72433 (17)	0.41278 (15)	0.0280 (5)	
H19	0.2665	0.7816	0.4325	0.034*	
C20	0.34886 (14)	0.69949 (17)	0.43800 (14)	0.0253 (5)	
H20	0.3921	0.7402	0.4744	0.030*	
C21	0.36103 (13)	0.61531 (17)	0.41050 (13)	0.0221 (5)	
H21	0.4124	0.5984	0.4279	0.027*	
C22	0.25177 (12)	0.35735 (15)	0.32766 (13)	0.0171 (4)	
C23	0.24544 (13)	0.36231 (17)	0.39383 (14)	0.0223 (5)	
H23	0.2734	0.4097	0.4356	0.027*	
C24	0.19816 (14)	0.29762 (18)	0.39790 (15)	0.0265 (5)	
H24	0.1933	0.3011	0.4424	0.032*	
C25	0.15793 (14)	0.22790 (18)	0.33766 (16)	0.0281 (5)	
H25	0.1248	0.1846	0.3404	0.034*	
C26	0.16576 (15)	0.22106 (18)	0.27322 (16)	0.0287 (5)	
H26	0.1388	0.1725	0.2324	0.034*	
C27	0.21322 (13)	0.28556 (16)	0.26844 (14)	0.0230 (5)	
H27	0.2193	0.2806	0.2248	0.028*	
Br1	0.456153 (14)	0.381335 (18)	0.588418 (14)	0.02564 (7)	
O3	0.5000	0.49982 (19)	0.7500	0.0300 (5)	
H3O	0.485 (2)	0.455 (3)	0.702 (2)	0.090 (14)*	

### Atomic displacement parameters (Å^2^)

**Table:**

	*U*^11^	*U*^22^	*U*^33^	*U*^12^	*U*^13^	*U*^23^
P1	0.0136 (2)	0.0170 (3)	0.0155 (2)	−0.0005 (2)	0.0084 (2)	0.0006 (2)
C1	0.0142 (9)	0.0186 (10)	0.0169 (10)	−0.0006 (8)	0.0077 (8)	0.0010 (8)
C2	0.0129 (9)	0.0227 (11)	0.0153 (9)	0.0020 (8)	0.0064 (8)	0.0026 (8)
C3	0.0206 (11)	0.0213 (11)	0.0230 (11)	−0.0003 (9)	0.0146 (9)	0.0007 (9)
C4	0.0208 (11)	0.0208 (11)	0.0260 (11)	−0.0012 (9)	0.0144 (10)	−0.0006 (9)
C5	0.0146 (10)	0.0261 (12)	0.0147 (10)	0.0029 (8)	0.0053 (8)	−0.0020 (8)
C6	0.0200 (11)	0.0386 (14)	0.0201 (10)	0.0080 (10)	0.0140 (9)	0.0060 (10)
C7	0.0208 (11)	0.0294 (13)	0.0227 (11)	0.0054 (10)	0.0132 (9)	0.0101 (10)
C8	0.0173 (11)	0.0318 (13)	0.0184 (11)	0.0062 (10)	0.0047 (9)	−0.0048 (9)
O1	0.0263 (9)	0.0428 (11)	0.0333 (9)	0.0090 (8)	0.0176 (8)	−0.0058 (8)
O2	0.0256 (9)	0.0222 (9)	0.0494 (11)	0.0018 (7)	0.0189 (9)	−0.0084 (8)
C9	0.0380 (16)	0.0257 (14)	0.078 (2)	0.0020 (12)	0.0304 (16)	−0.0167 (15)
C10	0.0177 (10)	0.0144 (10)	0.0174 (10)	0.0010 (8)	0.0100 (9)	0.0024 (8)
C11	0.0197 (11)	0.0266 (12)	0.0195 (10)	0.0039 (9)	0.0121 (9)	0.0022 (9)
C12	0.0204 (11)	0.0298 (13)	0.0185 (10)	0.0031 (10)	0.0077 (9)	0.0019 (9)
C13	0.0305 (12)	0.0228 (12)	0.0183 (10)	0.0019 (10)	0.0143 (10)	0.0027 (9)
C14	0.0291 (12)	0.0274 (12)	0.0231 (11)	−0.0023 (10)	0.0190 (10)	0.0021 (9)
C15	0.0185 (10)	0.0236 (12)	0.0204 (10)	−0.0018 (9)	0.0109 (9)	0.0011 (9)
C16	0.0186 (10)	0.0171 (10)	0.0188 (10)	0.0007 (8)	0.0110 (9)	0.0012 (8)
C17	0.0184 (11)	0.0266 (13)	0.0435 (15)	−0.0029 (10)	0.0156 (11)	−0.0082 (11)
C18	0.0274 (13)	0.0282 (14)	0.0588 (18)	0.0024 (11)	0.0283 (13)	−0.0050 (12)
C19	0.0387 (14)	0.0191 (12)	0.0342 (13)	0.0025 (10)	0.0258 (12)	−0.0011 (10)
C20	0.0260 (12)	0.0220 (12)	0.0203 (11)	−0.0020 (9)	0.0094 (10)	−0.0039 (9)
C21	0.0181 (10)	0.0233 (11)	0.0205 (10)	0.0016 (9)	0.0088 (9)	−0.0008 (9)
C22	0.0144 (10)	0.0180 (11)	0.0182 (10)	−0.0003 (8)	0.0092 (8)	0.0013 (8)
C23	0.0215 (11)	0.0256 (12)	0.0212 (11)	−0.0001 (9)	0.0134 (9)	0.0005 (9)
C24	0.0284 (12)	0.0319 (13)	0.0268 (12)	0.0044 (10)	0.0205 (10)	0.0072 (10)
C25	0.0282 (12)	0.0236 (12)	0.0413 (14)	−0.0011 (10)	0.0255 (12)	0.0058 (10)
C26	0.0308 (13)	0.0243 (12)	0.0350 (13)	−0.0084 (10)	0.0217 (11)	−0.0059 (10)
C27	0.0258 (12)	0.0223 (12)	0.0238 (11)	−0.0034 (9)	0.0162 (10)	−0.0020 (9)
Br1	0.02436 (12)	0.03282 (14)	0.02411 (12)	−0.00150 (10)	0.01670 (10)	0.00167 (10)
O3	0.0276 (13)	0.0313 (14)	0.0331 (13)	0.000	0.0190 (11)	0.000

### Geometric parameters (Å, °)

**Table:**

P1—C22	1.795 (2)	C12—H12	0.9500
P1—C16	1.799 (2)	C13—C14	1.383 (3)
P1—C10	1.800 (2)	C13—H13	0.9500
P1—C1	1.806 (2)	C14—C15	1.399 (3)
C1—C2	1.508 (3)	C14—H14	0.9500
C1—H1A	0.9900	C15—H15	0.9500
C1—H1B	0.9900	C16—C17	1.397 (3)
C2—C3	1.395 (3)	C16—C21	1.397 (3)
C2—C7	1.397 (3)	C17—C18	1.384 (4)
C3—C4	1.385 (3)	C17—H17	0.9500
C3—H3	0.9500	C18—C19	1.381 (4)
C4—C5	1.393 (3)	C18—H18	0.9500
C4—H4	0.9500	C19—C20	1.379 (4)
C5—C6	1.390 (3)	C19—H19	0.9500
C5—C8	1.495 (3)	C20—C21	1.388 (3)
C6—C7	1.388 (3)	C20—H20	0.9500
C6—H6	0.9500	C21—H21	0.9500
C7—H7	0.9500	C22—C27	1.391 (3)
C8—O1	1.207 (3)	C22—C23	1.399 (3)
C8—O2	1.343 (3)	C23—C24	1.384 (3)
O2—C9	1.454 (3)	C23—H23	0.9500
C9—H9A	0.9800	C24—C25	1.383 (4)
C9—H9B	0.9800	C24—H24	0.9500
C9—H9C	0.9800	C25—C26	1.387 (4)
C10—C15	1.388 (3)	C25—H25	0.9500
C10—C11	1.401 (3)	C26—C27	1.392 (3)
C11—C12	1.390 (3)	C26—H26	0.9500
C11—H11	0.9500	C27—H27	0.9500
C12—C13	1.389 (3)	O3—H3O	1.03 (4)
			
C22—P1—C16	107.00 (11)	C13—C12—H12	119.7
C22—P1—C10	108.96 (10)	C11—C12—H12	119.7
C16—P1—C10	109.85 (10)	C14—C13—C12	120.1 (2)
C22—P1—C1	112.43 (10)	C14—C13—H13	120.0
C16—P1—C1	107.06 (10)	C12—C13—H13	120.0
C10—P1—C1	111.41 (10)	C13—C14—C15	119.8 (2)
C2—C1—P1	116.34 (15)	C13—C14—H14	120.1
C2—C1—H1A	108.2	C15—C14—H14	120.1
P1—C1—H1A	108.2	C10—C15—C14	120.2 (2)
C2—C1—H1B	108.2	C10—C15—H15	119.9
P1—C1—H1B	108.2	C14—C15—H15	119.9
H1A—C1—H1B	107.4	C17—C16—C21	119.7 (2)
C3—C2—C7	118.9 (2)	C17—C16—P1	119.02 (17)
C3—C2—C1	120.7 (2)	C21—C16—P1	121.27 (17)
C7—C2—C1	120.3 (2)	C18—C17—C16	119.7 (2)
C4—C3—C2	120.7 (2)	C18—C17—H17	120.2
C4—C3—H3	119.7	C16—C17—H17	120.2
C2—C3—H3	119.7	C19—C18—C17	120.5 (2)
C3—C4—C5	120.4 (2)	C19—C18—H18	119.8
C3—C4—H4	119.8	C17—C18—H18	119.8
C5—C4—H4	119.8	C20—C19—C18	120.1 (2)
C6—C5—C4	119.1 (2)	C20—C19—H19	119.9
C6—C5—C8	118.7 (2)	C18—C19—H19	119.9
C4—C5—C8	122.2 (2)	C19—C20—C21	120.4 (2)
C7—C6—C5	120.7 (2)	C19—C20—H20	119.8
C7—C6—H6	119.7	C21—C20—H20	119.8
C5—C6—H6	119.7	C20—C21—C16	119.6 (2)
C6—C7—C2	120.2 (2)	C20—C21—H21	120.2
C6—C7—H7	119.9	C16—C21—H21	120.2
C2—C7—H7	119.9	C27—C22—C23	120.0 (2)
O1—C8—O2	123.9 (2)	C27—C22—P1	121.14 (17)
O1—C8—C5	124.3 (2)	C23—C22—P1	118.82 (16)
O2—C8—C5	111.8 (2)	C24—C23—C22	119.4 (2)
C8—O2—C9	116.1 (2)	C24—C23—H23	120.3
O2—C9—H9A	109.5	C22—C23—H23	120.3
O2—C9—H9B	109.5	C25—C24—C23	120.5 (2)
H9A—C9—H9B	109.5	C25—C24—H24	119.7
O2—C9—H9C	109.5	C23—C24—H24	119.7
H9A—C9—H9C	109.5	C24—C25—C26	120.2 (2)
H9B—C9—H9C	109.5	C24—C25—H25	119.9
C15—C10—C11	119.9 (2)	C26—C25—H25	119.9
C15—C10—P1	120.68 (16)	C25—C26—C27	119.8 (2)
C11—C10—P1	119.38 (17)	C25—C26—H26	120.1
C12—C11—C10	119.5 (2)	C27—C26—H26	120.1
C12—C11—H11	120.3	C22—C27—C26	119.9 (2)
C10—C11—H11	120.3	C22—C27—H27	120.1
C13—C12—C11	120.5 (2)	C26—C27—H27	120.1
			
C22—P1—C1—C2	54.37 (19)	C11—C10—C15—C14	−0.1 (3)
C16—P1—C1—C2	171.59 (16)	P1—C10—C15—C14	−176.59 (18)
C10—P1—C1—C2	−68.29 (19)	C13—C14—C15—C10	0.0 (4)
P1—C1—C2—C3	−71.4 (2)	C22—P1—C16—C17	−43.1 (2)
P1—C1—C2—C7	113.4 (2)	C10—P1—C16—C17	75.1 (2)
C7—C2—C3—C4	−1.9 (3)	C1—P1—C16—C17	−163.83 (19)
C1—C2—C3—C4	−177.2 (2)	C22—P1—C16—C21	136.78 (19)
C2—C3—C4—C5	1.1 (3)	C10—P1—C16—C21	−105.09 (19)
C3—C4—C5—C6	1.5 (3)	C1—P1—C16—C21	16.0 (2)
C3—C4—C5—C8	179.7 (2)	C21—C16—C17—C18	−0.8 (4)
C4—C5—C6—C7	−3.1 (3)	P1—C16—C17—C18	179.1 (2)
C8—C5—C6—C7	178.7 (2)	C16—C17—C18—C19	−0.3 (4)
C5—C6—C7—C2	2.2 (3)	C17—C18—C19—C20	1.2 (4)
C3—C2—C7—C6	0.3 (3)	C18—C19—C20—C21	−1.0 (4)
C1—C2—C7—C6	175.6 (2)	C19—C20—C21—C16	−0.1 (4)
C6—C5—C8—O1	11.6 (3)	C17—C16—C21—C20	1.0 (3)
C4—C5—C8—O1	−166.6 (2)	P1—C16—C21—C20	−178.90 (17)
C6—C5—C8—O2	−169.47 (19)	C16—P1—C22—C27	144.04 (18)
C4—C5—C8—O2	12.3 (3)	C10—P1—C22—C27	25.3 (2)
O1—C8—O2—C9	0.5 (3)	C1—P1—C22—C27	−98.69 (19)
C5—C8—O2—C9	−178.4 (2)	C16—P1—C22—C23	−36.1 (2)
C22—P1—C10—C15	−140.05 (18)	C10—P1—C22—C23	−154.78 (17)
C16—P1—C10—C15	103.04 (19)	C1—P1—C22—C23	81.2 (2)
C1—P1—C10—C15	−15.4 (2)	C27—C22—C23—C24	−2.5 (3)
C22—P1—C10—C11	43.5 (2)	P1—C22—C23—C24	177.58 (17)
C16—P1—C10—C11	−73.5 (2)	C22—C23—C24—C25	0.6 (4)
C1—P1—C10—C11	168.08 (17)	C23—C24—C25—C26	1.2 (4)
C15—C10—C11—C12	−0.1 (3)	C24—C25—C26—C27	−1.1 (4)
P1—C10—C11—C12	176.46 (18)	C23—C22—C27—C26	2.6 (3)
C10—C11—C12—C13	0.3 (4)	P1—C22—C27—C26	−177.47 (18)
C11—C12—C13—C14	−0.4 (4)	C25—C26—C27—C22	−0.8 (4)
C12—C13—C14—C15	0.2 (4)		

### Hydrogen-bond geometry (Å, °)

**Table:**

*D*—H···*A*	*D*—H	H···*A*	*D*···*A*	*D*—H···*A*
O3—H3O···Br1	1.03 (4)	2.22 (4)	3.2308 (17)	169 (3)
